# Gene Regulation, Modulation, and Their Applications in Gene Expression Data Analysis

**DOI:** 10.1155/2013/360678

**Published:** 2013-03-13

**Authors:** Mario Flores, Tzu-Hung Hsiao, Yu-Chiao Chiu, Eric Y. Chuang, Yufei Huang, Yidong Chen

**Affiliations:** ^1^Department of Electrical and Computer Engineering, University of Texas at San Antonio, San Antonio, TX 78249, USA; ^2^Greehey Children's Cancer Research Institute, University of Texas Health Science Center at San Antonio, San Antonio, TX 78229, USA; ^3^Graduate Institute of Biomedical Electronics and Bioinformatics, National Taiwan University, Taipei, Taiwan; ^4^Department of Epidemiology and Biostatistics, University of Texas Health Science Center at San Antonio, San Antonio, TX 78229, USA

## Abstract

Common microarray and next-generation sequencing data analysis concentrate on tumor subtype classification, marker detection, and transcriptional regulation discovery during biological processes by exploring the correlated gene expression patterns and their shared functions. Genetic regulatory network (GRN) based approaches have been employed in many large studies in order to scrutinize for dysregulation and potential treatment controls. In addition to gene regulation and network construction, the concept of the network modulator that has significant systemic impact has been proposed, and detection algorithms have been developed in past years. Here we provide a unified mathematic description of these methods, followed with a brief survey of these modulator identification algorithms. As an early attempt to extend the concept to new RNA regulation mechanism, competitive endogenous RNA (ceRNA), into a modulator framework, we provide two applications to illustrate the network construction, modulation effect, and the preliminary finding from these networks. Those methods we surveyed and developed are used to dissect the regulated network under different modulators. Not limit to these, the concept of “modulation” can adapt to various biological mechanisms to discover the novel gene regulation mechanisms.

## 1. Introduction

With the development of microarray [1] and lately the next generation sequencing techniques [2], transcriptional profiling of biological samples, such as tumor samples [3–5] and samples from other model organisms, have been carried out in order to study sample subtypes at molecular level or transcriptional regulation during the biological processes [6–8]. While common data analysis methods employ hierarchical clustering algorithms or pattern classification to explore correlated genes and their functions, the genetic regulatory network (GRN) approaches were employed to scrutinize for dysregulation between different tumor groups or biological processes (see reviews [9–12]). 

 To construct the network, most of research is focused on methods based on gene expression data derived from high-throughput technologies by using metrics such as Pearson or Spearman correlation [13], mutual information [14], co-determination method [15, 16], Bayesian methods [17, 18], and probabilistic Boolean networks [19]. Recently, new transcriptional regulation via competitive endogenous RNA (ceRNAs) has been proposed [20, 21], introducing additional dimension in modeling gene regulation. This type of regulation requires the knowledge of microRNA (miRNA) binding targets [22, 23] and the hypothesis of RNA regulations via competition of miRNA binding. Common GRN construction tries to confine regulators to be transcription factor (TF) proteins, a primary transcription programming machine, which relies on sequence-specific binding sites at target genes' promoter regions. In contrast, ceRNAs mediate gene regulation via competing miRNAs binding sites in target 3′UTR region, which exist in >50% of mRNAs [22, 24]. In this study, we will extend the current network construction methods by incorporating regulation via ceRNAs.

 In tumorigenesis, gene mutation is the main cause of the cancer [25]. The mutation may not directly reflect in the change at the gene expression level; however, it will disrupt gene regulation [26–28]. In Hudson et al., they found that mutated myostatin and MYL2 showed different coexpressions when comparing to wild-type myostatin. Chun et al. also showed that oncogenic KRAS modulates HIF-1*α* and HIF-2*α* target genes and in turn modulates cancer metabolism. Stelniec-Klotz et al. presented a complex hierarchical model of KRAS modulated network followed by double perturbation experiments. Shen et al. [29] showed a temporal change of GRNs modulated after the estradiol stimulation, indicating important role of estrogen in modulating GRNs. Functionally, modulation effect of high expression of *ESR1* was also reported by Wilson and Dering [30] where they studied previously published microarray data with cells treated with hormone receptor agonists and antagonists [31–33]. In this study, a comprehensive review of existing algorithms to uncover the modulators was provided. Given either mutation or protein expression status was unknown under many of reported studies, the problem of how to partition the diverse samples with different conditions, such as active or inactive oncogene status (and perhaps a combination of multiple mutations), and the prediction of a putative modulator of gene regulation remains a difficult task.

 By combining gene regulation obtained from coexpression data and ceRNAs, we report here an early attempt to unify two systems mathematically while assuming a known modulator, estrogen receptor (ER). By employing the TCGA [3] breast tumor gene expressions data and their clinical test result (ER status), we demonstrate the approach of obtaining GRN via ceRNAs and a new presentation of ER modulation effects. By integrating breast cancer data into our unique ceRNAs discovery website, we are uniquely positioned to further explore the ceRNA regulation network and further develop the discovery algorithms in order to detect potential modulators of regulatory interactions.

## 2. Models of Gene Regulation and Modulation

### 2.1. Regulation of Gene Expression

**Figure 1:**
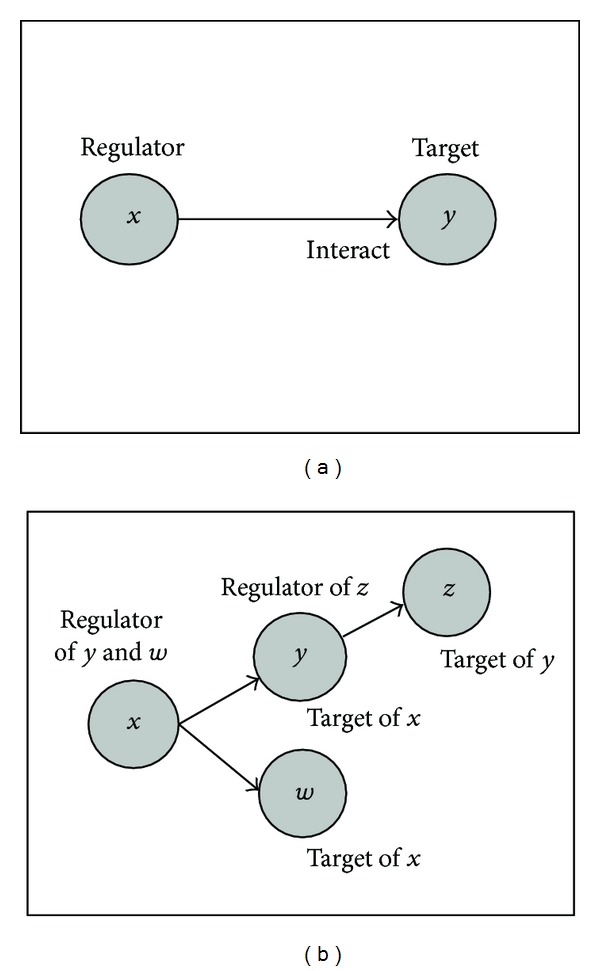
Regulator-target pair in genetic regulatory network model: (a) basic regulator-target pair and (b) regulator-target complex.

The complex relationships among genes and their products in a cellular system can be studied using genetic regulatory networks (GRNs). The networks model the different states or phenotypes of a cellular system. In this model, the interactions are commonly modeled as regulator-target pairs with edges between regulator and target pair representing their interaction direction, as shown in Figure 1(a). In this model a target gene is a gene whose expression can be altered (activated or suppressed) by a regulator gene. This definition of a target gene implies that any gene can be at some point a target gene or a direct or indirect regulator depending on its position in the genetic regulatory network. The regulator gene is a gene that controls (activates or suppresses) its target genes' expression. The consequences of these activated (or suppressed) genes sometimes are involved in specific biological functions, such as cell proliferation in cancer. Examples of regulator-target pair in biology are common. For example, a target gene CDCA7 (cell division cycle-associated protein 7) is a c-Myc (regulator) responsive gene, and it is part of c-Myc-mediated transformation of lymphoblastoid cells. Furthermore, as shown in Figure 1(b), a regulator gene can also act as a target gene if there exists an upstream regulator.

  If the interaction is modeled after Boolean network (BN) model [34], then

(1) $$\begin{array}{c} y_{i}\left(t+1\right)=f_{i}\left(x_{j_{1}}\left(t\right),\ldots ,{x_{j}}_{k}\left(t\right),y_{i}\left(t\right)\right), \end{array}$$

where each regulator *x*_*j*_ ∈ {0,1} is a binary variable, as well as it is target *y*_*i*_. As described by (1), the target *y*_*i*_ at time *t* + 1 is completely determined by the values of its regulators at time *t* by means of a Boolean function *f*_*i*_ ∈ *F*, where *F* is a collection of Boolean functions. Thus, the Boolean network *G*(*V*, *F*) is defined as a set of nodes (genes) *V* = {*x*_1_, *x*_2_,…, *x*_*n*_} and a list of functions (edges or interactions) *F* = {*f*_1_, *f*_2_, …, *f*_*n*_}. Similarly such relationship can be defined in the framework of Bayesian network where the similar regulators-target relationship as defined in (1) can be modeled by the distribution

(2) P(yi(t+1),xj1(t),…,xjk(t),yi(t))  =P(yi(t+1) ∣ Parents(yi(t+1)))   ×P(Parents(yi(t+1))),

where Parents(*y*_*i*_(*t* + 1)) = {*x*_*j*_1__(*t*),…,*x*_*j*__*k*_(*t*), *y*_*i*_(*t*)} is the set of regulators, or parents, of *y*_*i*_, *P*(*y*_*i*_(*t* + 1) | Parents  (*y*_*i*_(*t* + 1))) is the conditional distribution defining the regulator-target relationship, and *P*(Parents(*y*_*i*_(*t* + 1))) models the prior distribution of regulators. Unlike in (1), the target and regulators in (2) are modeled as random variables. Despite of this difference, in both (1) and (2), the target is always a function (or conditional distribution) of the regulator (or parents). When the relationship is defined by a Boolean function as in (1), the conditional distribution in (2) take the form of a binomial distribution (or a multinomial distribution when both regulators and target take more than two states). Other distributions such as the Gaussian and Poisson can be introduced to model more complex relationships than the Boolean. The network construction, inference, and control, however, are beyond the scope of this paper, and we leave the topics to the literatures [9, 35, 36].

**Figure 2:**
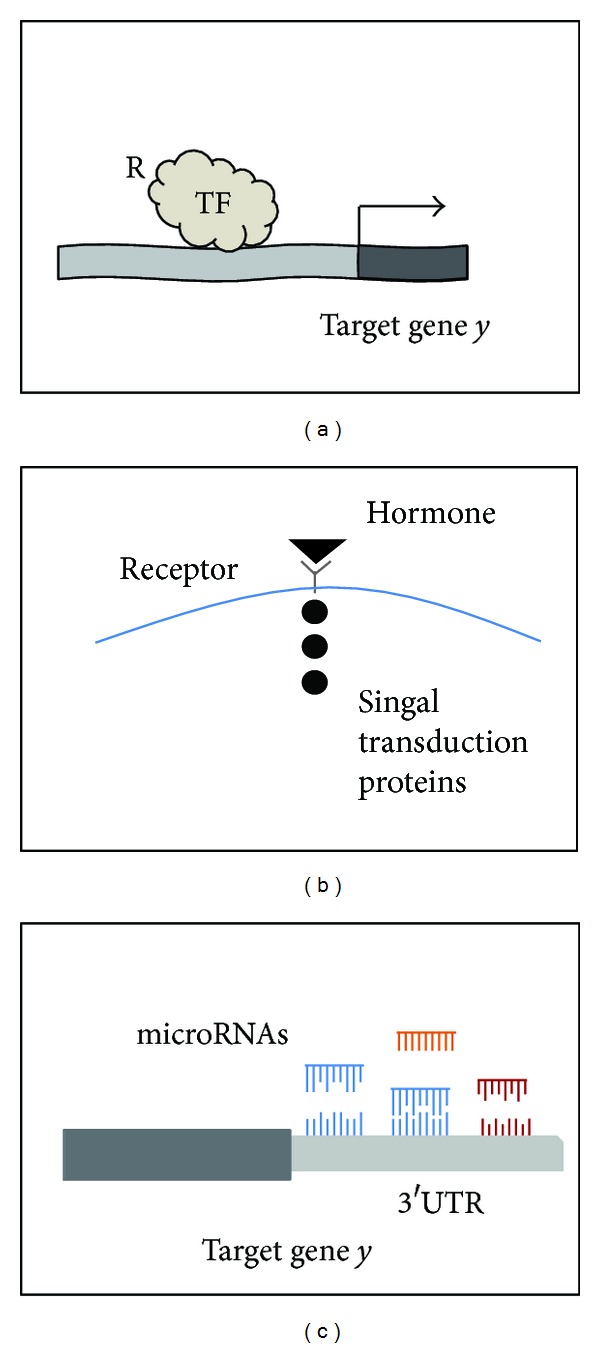
Three different cases of regulation of gene expression that share the network representation of a regulator target interaction.

 The interactions among genes and their products in a complex cellular process of gene expression are diverse, governed by the central dogma of molecular biology [37]. There are different regulation mechanisms that can actuate during different stages.  Figure 2 shows three different cases of regulation of gene expression.  Figure 2(a) shows the case of regulation of expression in which a transcription factor (TF) regulates the expression of a protein-coding gene (in dark grey) by binding to the promoter region of target gene *y*.Figure 2(b) is the case of regulation at the protein level in which a ligand protein interacts with a receptor to activate relay molecules to transduce outside signals directly into cell behavior.  Figure 2(c) is the case of regulation at the RNA level in which one or more miRNAs regulate target mRNA *y* by translational repression or target transcript degradation via binding to sequence-specific binding sites (called miRNA response elements or MREs) in 3′UTR region. As illustrated in Figure 2(c), the target genes/proteins all contain a domain of binding or docking site, enabling specific interactions between regulator-target pairs, a common element in network structure. 

### 2.2. Modulation of Gene Regulation

**Figure 3:**
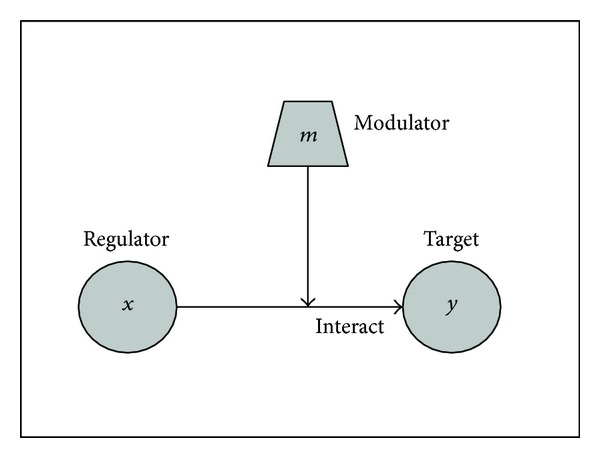
Graphical representation of the triplet interaction of regulator *x*, target *y*, and modulator *m*.

Different from the concept of a coregulator commonly referred in the regulatory biology, a modulator denotes a gene or protein that is capable of altering the endogenous gene expression at one stage or time. In the context of this paper, we specifically define a modulator to be a gene that can systemically influence the interaction of regulator-target pair, either to activate or suppress the interaction in the presence/absence of the modulator. One example of modulator is the widely studied estrogen receptor (ER) in breast cancer studies [38–40]; the ER status determines not only the tumor progression, but also the chemotherapy treatment outcomes. It is well known that binding of estrogen to receptor facilitates the ER activities to activate or repress gene expression [41], thus effectively modulating the GRN.  Figure 3 illustrates the model of the interaction between a modulator (*m*) and a regulator (*x*) target (*y*) pair that it modulates. 

 Following the convention used in (1) and (2), the modulation interaction in Figure 3 can be modeled by

(3) $$\begin{array}{c} y=\;{ℱ}^{\left(m\right)}\left(x\right), \end{array}$$

where *y* represents target expression, *x* represents the parents (regulators) of target *y*, and *ℱ* ^(*m*)^(·) is the regulation function modulated by *m*. When *ℱ* ^(*m*)^(·) is stochastic, the relationship is modeled by the conditional distribution as

(4) $$\begin{array}{c} p\left(y,x\;|\;m\right)=\;p\left(y\;|\;x,m\right)p\left(x\;|\;m\right), \end{array}$$

where *p*(*y* | *x*, *m*) models the regulator-target relationship modulated by *m* and *p*(*x* | *m*) defines the prior distribution of regulators (parents) expression modulated by *m*. Different distribution models can be used to model different mechanisms for modulation. At the biological level, there are different mechanisms for modulation of the interaction *x*-*y*, and currently several algorithms for prediction of the modulators has been developed. This survey presents the latest formulations and algorithms for prediction of modulators. 

## 3. Survey of Algorithms of Gene Regulation and Modulation Discovery

During the past years, many computational tools have been developed for regulation network construction, and then depending on the hypothesis, modulator concept can be tested and extracted. Here we will focus on modulator detection algorithms (MINDy, Mimosa, GEM, and Hermes). To introduce gene-gene interaction concept, we will also briefly discuss algorithms for regulation network construction (ARACNE) and ceRNA identification algorithm (MuTaMe). 

### 3.1. ARACNE (Algorithm for the Reconstruction of Accurate Cellular Networks)

 ARACNE [14, 42] is an algorithm that extracts transcriptional networks from microarray data by using an information-theoretic method to reduce the indirect interactions. ARACNE assumes that it is sufficient to estimate 2-way marginal distributions, when sample size *M* > 100, in genomics problems, such that

(5) $$\begin{array}{c} p\left(x_{i}\right)=\frac{1}{z}e^{-[\sum_{i=1}^{N}\phi_{i}(x_{i})+\sum_{i,j}^{N}\phi_{ij}(x_{i},y_{j})]}. \end{array}$$

Or a candidate interaction can be identified using estimation of mutual information MI of genes *x* and *y*, MI(*x*, *y*) = MI_*xy*_, where MI_*xy*_ = 1 if genes *x* and *y* are identical, and MI_*xy*_ is zero if *p*(*x*, *y*) = *p*(*x*)*p*(*y*), or *x* and *y* are statistically independent. Specifically, the estimation of mutual information of gene expressions *x* and *y* of regulator and target genes is done by using the Gaussian kernel estimator. The ARACNE takes additional two steps to clean the network: (1) removing MI if its *P* value is less than that derived from two independent genes via random permutation and (2) data processing inequality (DPI). The algorithm further assumes that for a triplet gene (*g*_*x*_, *g*_*y*_, *g*_*z*_), where *g*_*x*_ regulates *g*_*z*_, through *g*_*y*_, then

(6) MIx,z<min⁡(MIx,y,MIy,z), if  x→y→z,         with  no  alternative  path,

where → represents regulation relationship. In other words, the lowest mutual information MI_*x*,*z*_ is from an indirect interaction and thus shall be removed from the GRN by ARACNE in the DPI step. A similar algorithm was proposed [43] to utilize conditional mutual information to explore more than 2 regulators. 

### 3.2. MINDy (Modulator Inference by Network Dynamics)

 Similar to ARACNE, MINDy is also an information-theoretic algorithm [44]. However, MINDy aims to identify potential transcription factor-(TF-target) gene pairs that can be modulated by a candidate modulator. MINDy assumes that the expressions of the modulated TF-target pairs are of different correlations under different expression state of the modulator. For simplicity and computational consideration, MINDy considers only two modulator expression states, that is, up- (*m* = 1) or down-expression (*m* = 0). Then, it tests if the expression correlations of potential TF-target pairs are significantly different for modulator up-expression versus down-expression. The modulator dependent correlation is assessed by the conditional mutual information (CMI) or *I*(*x*, *y* | *m* = 0) and *I*(*x*, *y* | *m* = 1). Similar to ARACNE, the CMI is calculated using the Gaussian kernel estimator. To test if a pair of TF (*y*) and target (*x*) is modulated by *m*, the CMI difference can be calculated as

(7) $$\begin{array}{c} \Delta I=I\left(x,y\;|\;m=1\right)-I\left(x,y\;|\;m=0\right). \end{array}$$

The pair is determined to be modulated if Δ*I* ≠ 0. The significance *P* values for Δ*I* ≠ 0 is computed using permutation tests. 

### 3.3. Mimosa

Similarly to MINDy, Mimosa [45] was proposed to identify modulated TF-target pairs. However, it does not preselect a set of modulators of interest but rather aims to also search for the modulators. Mimosa also assumes that a modulator takes only two states, that is, absence and presence or 0 and 1. The modulated regulator-target pair is further assumed to be correlated when a modulator is present but uncorrelated when it is absent. Therefore, the distribution of a modulated TF-target pair, *x* and *y*, naturally follows a mixture distribution

(8) $$\begin{array}{c} p\left(x,y\right)=\;\pi p\left(x,y\;|\;m=0\right)+\left(1-\pi \right)p\left(x,ym=1\right), \end{array}$$

where *π* is the probability of the modulator being absent. Particularly, an uncorrelated and correlated bivariant Gaussian distributions were introduced to model different modulated regulator-target relationship, such that

(9a) $$\begin{array}{c} p\left(x,y\;|\;m=0\right)=\frac{1}{2\pi }e^{-(1/2)(x^{2}+y^{2})}, \end{array}$$

(9b) p(x,y ∣ m=1) =12π1−α2e  −(1/2)(x2+y2+2αxy)/(1−α2),

where *α* models the correlation between *x* and *y* when the modulator is present. With this model, Mimosa sets out to fit the samples of every pair of potential regulator target with the mixture model (7). This is equivalent to finding a partition of the paired expression samples into the correlated and uncorrelated samples. The paired expression samples that possess such correlated-uncorrelated partition (0.3 < *π* < 0.7  and | *α* | >0.8) are determined to be modulated. To identify the modulator of a (or a group of) modulated pair(s), a weighted *t*-test was developed to search for the genes whose expressions are differentially expressed in the correlated partition versus the uncorrelated partition. 

### 3.4. GEM (Gene Expression Modulator)

 GEM [46] improves over MINDy by predicting how a modulator-TF interaction affects the expression of the target gene. It can detect new types of interactions that result in stronger correlation but low Δ*I*, which therefore would be missed by MINDy. GEM hypothesizes that the correlation between the expression of a modulator *m* and a target *x* must change, as that of the TF *x* changes. Unlike the previous surveyed algorithms, GEM first transforms the continuous expression levels to binary states (up- (1) or down-expression (0)) and then works only with discrete expression states. To model the hypothesized relationship, the following model is proposed:

(10) $$\begin{array}{c} P\left(x=1\;|\;y,m\right)=\alpha_{c}+\alpha_{m}m+\alpha_{y}y+\gamma my, \end{array}$$

where *α*_*c*_ is a constant, *α*_*m*_ and *α*_*y*_ model the effect of modulator and TF on the target genes, and *γ* represents the effect of modulator-TF interaction on the target gene. If the modulator-TF interaction has an effect on *x*, then *γ* will be nonzero. For a given (*x*, *y*, *m*) triplets GEM devised an algorithm to estimate the model coefficients in (10) and a test to determine if *γ* is nonzero, or *m* is a modulator of *x* and *y*. 

### 3.5. MuTaMe (Mutually Targeted MRE Enrichment)

**Figure 4:**
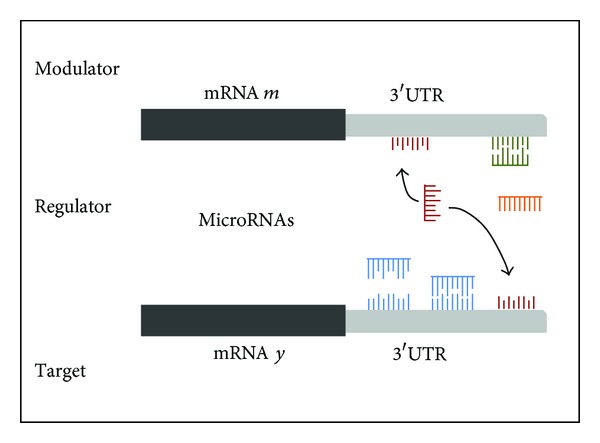
Modulation of gene regulation by competing mRNAs.

 The goal of MuTaMe [21] is to identify ceRNA networks of a gene of interest (GoI) or mRNA that share miRNA response elements (MREs) of same miRNAs.  Figure 4 shows two mRNAs, where one is the GoIy and the other is a candidate ceRNA or modulator *m*. In the figure, the miRNA represented in color red has MREs in both mRNA *y* and mRNA *m*; in this case the presence of mRNA *m* will start the competition with *y* for miRNA represented in color red.

 The hypothesis of MuTaMe is that mRNAs that have many of the same MREs can regulate each other by competing for miRNAs binding. The input of this algorithm is a GoI, which is targeted by a group of miRNAs known to the user. Then, from a database of predicted MREs for the entire transcriptome, it is possible to obtain the binding sites and its predicted locations in the 3′UTR for all mRNAs. This data is used to generate scores for each mRNA based on several features:

- the number of miRNAs that an mRNA *m* shares with the GoI *y*;
- the density of the predicted MREs for the miRNA; it favors the cases in which more MREs are located in shorter distances;
- the distribution of the MREs for every miRNA; it favors situations in which the MREs tend to be evenly distributed;
- the number of MREs predicted to target *m*; it favors situations where each miRNA contains more MREs in *m*.

Then each candidate transcript *m* will be assigned a score that results from multiplying the scores in (a) to (d). This score indicates the likelihood of the candidates to be ceRNAs and will be used to predict ceRNAs. 

### 3.6. Hermes

Hermes [20] is an extension of MINDy that infers candidate modulators of miRNA activity from expression profiles of genes and miRNAs of the same samples. Hermes makes inferences by estimating the MI and CMI. However, different from MINDy (7), Hermes extracts the dependences of this triplet by studying the difference between the CMI of *x* expression and *y* expression conditional on the expression of *m* and the MI of *x* and *y* expressions as follows:

(11) $$\begin{array}{c} I=I\left(x;y\;|\;m\right)-I\left(x;y\right). \end{array}$$

These quantities and their associated statistical significance can be computed from collections of expression of genes with number of samples 250 or greater. Hermes expands MINDy by providing the capacity to identify candidate modulator genes of miRNAs activity. The presence of these modulators (*m*) will affect the relation between the expression of the miRNAs targeting a gene (*x*) and the expression level of this gene (*x*).

 In summary, we surveyed some of the most popular algorithms for the inference of modulator. Additional modulator identification algorithms are summarized in Table 1. It is worth noting that the concept of modulator applies to cases beyond discussed in this paper. Such example includes the multilayer integrated regulatory model proposed in Yan et al. [49], where the top layer of regulators could be also considered as “modulators.”

## 4. Applications to Breast Cancer Gene Expression Data

Algorithms of utilizing modulator concept have been implemented in various software packages. Here we will discuss two new applications, MEGRA and TraceRNA, implemented in-house specifically to utilize the concept of differential correlation coefficients and ceRNAs to construct a modulated GRN with a predetermined modulator. In the case of MGERA, we chose estrogen receptor, *ESR1*, as the initial starting point, since it is one of the dominant and systemic factor in breast cancer; in the case of TraceRNA, we also chose gene *ESR1* and its modulated gene network. Preliminary results of applications to TCGA breast cancer data are reported in the following 2 sections.

### 4.1. MGERA

The Modulated Gene Regulation Analysis algorithm (MGERA) was designed to explore gene regulation pairs modulated by the modulator *m*. The regulation pairs can be identified by examining the coexpression of two genes based on Pearson correlation (similar to (7) in the context of correlation coefficient). Fisher transformation is adopted to normalize the correlation coefficients biased by sample sizes to obtain equivalent statistical power among data with different sample sizes. Statistical significance of difference in the absolute correlation coefficients between two genes is tested by the student *t*-test following Fisher transformation. For the gene pairs with significantly different coefficients between two genes, active and deactive statuses are identified by examining the modulated gene expression pairs (MGEPs). The MGEPs are further combined to construct the *m* modulated gene regulation network for a systematic and comprehensive view of interaction under modulation.

**Figure 5:**
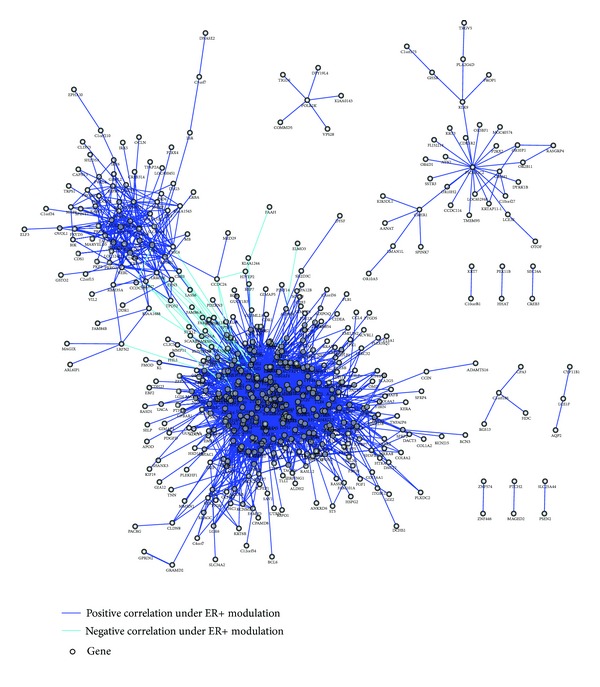
ER+ modulated gene regulation network.

 To demonstrate the ability of MGERA, we set estrogen receptor (ER) as the modulator and applied the algorithm to TCGA breast cancer expression data [3] which contains 588 expression profiles (461 ER+ and 127 ER−). By using *P* value <0.01 and the difference in the absolute Pearson correlation coefficients >0.6 as criteria, we identified 2,324 putative ER+ MGEPs, and a highly connected ER+ modulated gene regulation network was constructed (Figure 5). The top ten genes with highest connectivity was show in Table 2. The cysteine/tyrosine-rich 1 gene (*CYYR1*), connected to 142 genes, was identified as the top hub gene in the network and thus may serve as a key regulator under ER+ modulation. Gene Ontology analysis of *CYYR1* and its connected neighbor genes revealed significant association with extracellular matrix, epithelial tube formation, and angiogenesis.

### 4.2. TraceRNA

**Figure 6:**
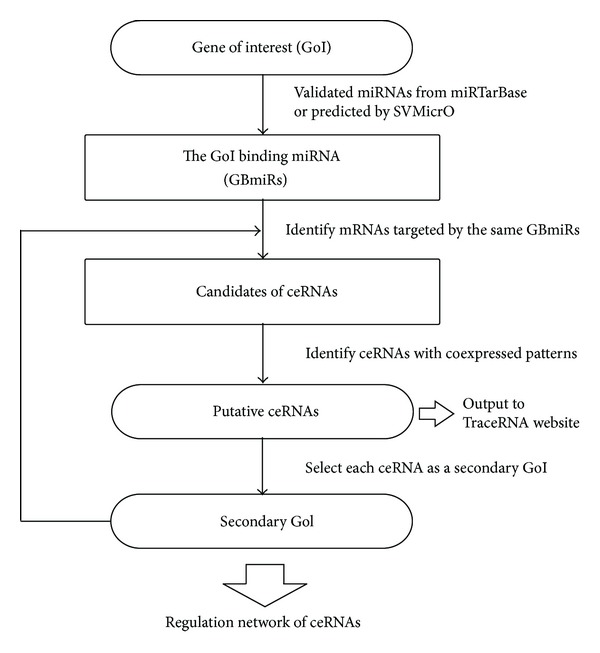
The analysis flow chart of TraceRNA.

To identify the regulation network of ceRNAs for a GoI, we developed a web-based application TraceRNA presented earlier in [50] with extension to regulation network construction. The analysis flow chart of TraceRNA was shown in Figure 6. For a selected GoI, the GoI binding miRNAs (GBmiRs) were derived either validated miRNAs from miRTarBase [51] or predicted miRNAs from SVMicrO [52]. Then mRNAs (other than the given GoI) also targeted by GBmiRs were identified as the candidates of ceRNAs. The relevant (or tumor-specific) gene expression data were used to further strengthen relationship between the ceRNA candidates and GoI. The candidate ceRNAs which coexpressed with GoI were reported as putative ceRNAs. To construct the gene regulation network via GBmiRs, we set each ceRNA as the secondary GoI, and the ceRNAs of these secondary GoIs were identified by applying the algorithm recursively. Upon identifying all the ceRNAs, the regulation network of ceRNAs of a given GoI was constructed.

**Figure 7:**
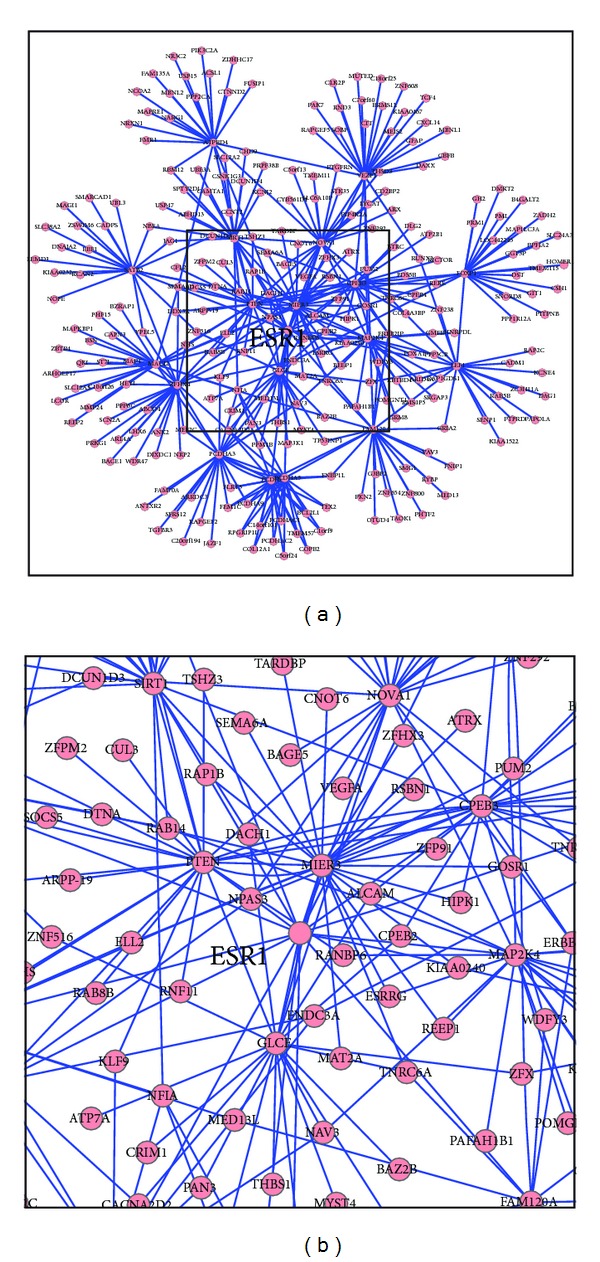
(a) ceRNA network for gene of interest ESR1 generated using TraceRNA. (b) Network graph enlarged at ESR1.

To identify ceRNA candidates, three miRNAs binding prediction algorithms, SiteTest, SVMicrO, and BCMicrO, were used in TracRNA. SiteTest is an algorithm similar to MuTaMe and uses UTR features for target prediction. SVMicrO [52] is an algorithm that uses a large number of sequence-level site as well as UTR features including binding secondary structure, energy, and conservation, whereas BCMicrO [53] employs a Bayesian approach that integrates predictions from 6 popular algorithms including TargetScan, miRanda, PicTar, mirTarget, PITA, and DIANA-microT. Pearson correlation coefficient was used to test the coexpression between the GoI and the candidate ceRNAs. We utilized TCGA breast cancer cohort [3] as the expression data, by using 60% of GBmiRs as common miRNAs and Pearson correlation coefficient >0.9 as criteria. The final scores of putative ceRNAs (see Table 3, last column) were generated by using Borda merging method which rerank the sum of ranks from both GBmiR binding and coexpression *P* values [54]. To illustrate the utility of the TraceRNA algorithm for breast cancer study, we also focus on the genes interacted with the estrogen receptor alpha, *ESR1*, with GBmiRs including *miR-18a, miR-18b, miR-193b, miR-19a, miR-19b, miR-206, miR-20b, miR-22, miR-221, miR-222, miR-29b, and miR-302c*. The regulation network generated by *ESR1* as the initial GoI is shown in Figure 7, and the top 18 ceRNAs are provided in Table 3. The TraceRNA algorithm can be accessed http://compgenomics.utsa.edu/cerna/.

## 5. Conclusions

In this report, we attempt to provide a unified concept of modulation of gene regulation, encompassing earlier mRNA expression based methods and lately the ceRNA method. We expect the integration of ceRNA concept into the gene-gene interactions, and their modulator identification will further enhance our understanding in gene interaction and their systemic influence. Applications provided here also represent examples of our earlier attempt to construct modulated networks specific to breast cancer studies. Further investigation will be carried out to extend our modeling to provide a unified understanding of genetic regulation in an altered environment.

## Figures and Tables

**Table 1:** Gene regulation network and modulator identification methods.

Algorithm	Features	References
ARACNE	Interaction network constructed via mutual information (MI).	[14, 42]
Network profiler	A varying-coefficient structural equation model (SEM) to represent the modulator-dependent conditional independence between genes.	[47]
MINDy	Gene-pair interaction dependency on modulator candidates by using the conditional mutual information (CMI).	[44]
Mimosa	Search for modulator by partition samples with a Gaussian mixture model.	[45]
GEM	A probabilistic method for detecting modulators of TFs that affect the expression of target gene by using a priori knowledge and gene expression profiles.	[46]
MuTaMe	Based on the hypothesis that shared MREs can regulate mRNAs by competing for microRNAs binding.	[21]
Hermes	Extension of MINDy to include microRNAs as candidate modulators by using CMI and MI from expression profiles of genes and miRNAs of the same samples.	[20]
ER*α* modulator	Analyzes the interaction between TF and target gene conditioned on a group of specific modulator genes via a multiple linear regression.	[48]

**Table 2:** Hub genes derived from modulated gene regulation network (Figure 5).

Gene	Number of ER+ MGEPs
CYYR1	142
MRAS	109
C9orf19	95
LOC339524	93
PLEKHG1	92
FBLN5	91
BOC	91
ANKRD35	89
FAM107A	83
C16orf77	73

**Table 3:** Top 18 candidate ceRNAs for *ESR1* as GOI obtained from TraceRNA. *ESR1* is at rank of 174 (not listed in this table).

Gene symbol	SVMicrO-based prediction		Expression correlation		Final score
	Score	*P* value	Score	*P* value
FOXP1	1.066	0.0043	0.508	0.016	1212
VEZF1	0.942	0.0060	0.4868	0.020	1179
NOVA1	0.896	0.0067	0.479	0.023	1160
CPEB3	0.858	0.0074	0.484	0.022	1149
MAP2K4	0.919	0.0064	0.322	0.097	1139
FAM120A	0.885	0.0069	0.341	0.082	1130
PCDHA3	0.983	0.0054	0.170	0.215	1125
SIRT1	0.927	0.0062	0.230	0.162	1117
PCDHA5	0.983	0.0054	0.148	0.233	1113
PTEN	0.898	0.0067	0.221	0.168	1104
PCDHA1	0.983	0.0054	0.140	0.239	1103
NBEA	0.752	0.0098	0.491	0.020	1102
ZFHX4	0.970	0.0056	0.154	0.229	1097
GLCE	0.798	0.0087	0.3231	0.096	1096
MAGI2	0.777	0.0092	0.321	0.097	1086
SATB2	0.801	0.0086	0.243	0.151	1078
LEF1	0.753	0.0098	0.291	0.112	1065
ATPBD4	0.819	0.0082	0.170	0.215	1060
